# Anti-*Helicobacter pylori* treatment can effectively improve the clinical remission rates of irritable bowel syndrome: a controlled clinical trial meta-analysis

**DOI:** 10.6061/clinics/2020/e1857

**Published:** 2020-11-02

**Authors:** Yan Xiong, Lulu Liu, Xuchun Zhou, Youfei Wen, Ruonan Wang

**Affiliations:** I Department of Gastroenterology, the First Affiliated Hospital of Chongqing Medical University, People’s Republic of China; II Hainan Medical College, People’s Republic of China

**Keywords:** Irritable Bowel Syndrome, *Helicobacter Pylori* Infection, IBS treatment, Meta-analysis

## Abstract

Here we used a meta-analysis of several clinical trials to determine whether anti-*Helicobacter pylori* therapy has any positive effect on IBS patients. Here we compared the effective clinical remission rates between IBS patients treated with anti-*H. pylori* therapy and those who were not. This data would provide more clinical evidence regarding the efficacy of novel treatments and intervention points for IBS patients.

Relevant studies were identified using keyword searches on various electronic databases, including PubMed, Embase, the Cochrane Central Register of Controlled Trials, CNKI, and CBM. Keywords included “*helicobacter pylori*” and “irritable bowel syndrome” among others. The literature was screened using relatively strict inclusion and exclusion criteria and RevMan 5.3.5 and Stata 15.1 software were used for meta-analysis and to assess publication bias and sensitivity.

A total of ten studies met all of the inclusion criteria; these included 655 IBS patients with *H. pylori* infection, of these, 385 patients were in the experimental group and 270 patients were in the control group. A random-effects model was used to pool the odds ratios (ORs) with a 95% confidence interval (CIs) and the combined OR was 2.87 (95% CI: 1.74-4.72), *p*<0.0001. These findings suggest that anti-*H. pylori* therapy can effectively improve the remission rates of *H. pylori-*positive IBS patients.

*H. pylori* infection is known to correlate with the incidence of IBS. Anti-*H. pylori* treatment can effectively improve the clinical remission rates of IBS patients. Whether this means that IBS patients should be actively treated with anti-*H. pylori* compounds as a novel strategy to improve the remission rates needs to be evaluated *in vivo*.

## INTRODUCTION

Irritable bowel syndrome (IBS) is a functional gastrointestinal disease with variable incidence rate in countries across the globe. Incomplete statistics suggest that the worldwide incidence of IBS ranges from 7% to 15% with this increasing to as much as 20% in some populations in China (1). IBS is characterized by recurrent abdominal pain and changes in bowel movements. Its influence on the quality of life cannot be ignored but clinical interventions remain largely ineffective (2). Studies have shown that IBS is a complex syndrome caused by long-term interactions between various psychosomatic factors, neuroendocrine factors, immunity, drugs, and infection, among others (3,4). *Helicobacter pylori*, an important gastroenterological pathogen has an infection rate of up to 50% in Chinese populations (5), and has been linked to a variety of digestive tract diseases. However, the belief that the *H. pylori* infection rate in IBS patients is higher than that of the healthy population is still controversial. This study was designed to evaluate meta-data from several clinical studies to identify any evidence supporting the positive relationship between anti-*H. pylori* therapy and improved clinical remission rates for IBS.

## MATERIAL AND METHODS

### Inclusion and exclusion criteria

**Inclusion criteria:** The following study criteria were used: (1) Study design: clinical controlled trial; (2) Study content: discussion of any differences in the effective remission rates of IBS patients with or without anti-*H. pylori* treatment; the specific sample size and effective remission rates for both the experimental and control groups must have been accurately recorded; (3) Study subjects: IBS patients who meet the Rome II, III, or IV diagnostic criteria and an *H. pylori-positive* result based on either a ^13^C, ^14^C urea breath test (UBT) or rapid urease test (RUT); (4) Study results: the strategy used for the evaluation of the effective remission rate for IBS must be similar between studies. Expected effect: abdominal pain, diarrhea, constipation, and other symptoms almost completely disappeared; Effective outcomes: abdominal pain, diarrhea, constipation and other symptoms are significantly reduced but still exist; Invalid: abdominal pain, diarrhea, constipation, and other symptoms were not reduced or patients experienced increased severity. Total effective rate = (number of obvious effect cases+number of effective cases)/number of total cases × 100%.

### Exclusion criteria

The following exclusion criteria were used: (1) Non-clinical data or repeated publications including reviews and animal studies; (2) Unavailable text, incomplete data, lack of control group data or incalculable data; (3) Articles using previously published data.

### Retrieval strategies

PubMed, Embase, the Cochrane Central Register of Controlled Trials, CNKI, CBM, Wanfang and VIP databases were searched using various search terms, including “*helicobacter pylori*,” “*H. pylori*,” “*Campylobacter pylori*,” “irritable bowel syndrome,” “Irritable Bowel Syndromes,” “Syndrome, Irritable Bowel,” “Syndromes, Irritable Bowel,” “Colon, Irritable,” “Irritable Colon,” “Colitis, Mucous,” “Colitides, Mucous,&quot; “Mucous Colitides,” and “Mucous Colitis.” PubMed database retrieval strategy: ((((((((((((Irritable Bowel Syndromes[Title/Abstract]) OR Syndrome, Irritable Bowel[Title/Abstract]) OR Syndromes, Irritable Bowel[Title/Abstract]) OR Colon, Irritable[Title/Abstract]) OR Irritable Colon[Title/Abstract]) OR Colitis, Mucous[Title/Abstract]) OR Colitides, Mucous[Title/Abstract]) OR Mucous Colitides[Title/Abstract]) OR Mucous Colitis[Title/Abstract])) OR irritable bowel syndrome[Mesh])) AND ((((Campylobacter pylori[Title/Abstract]) OR H.pylori[Title/Abstract])) OR helicobacter pylori[Mesh]). The search results were reviewed independently by two researchers and were evaluated by a third person if there were any objections or doubts. The search period covered nearly 15 years and ended in September 2019.

### Data extraction

**Figure 1:**
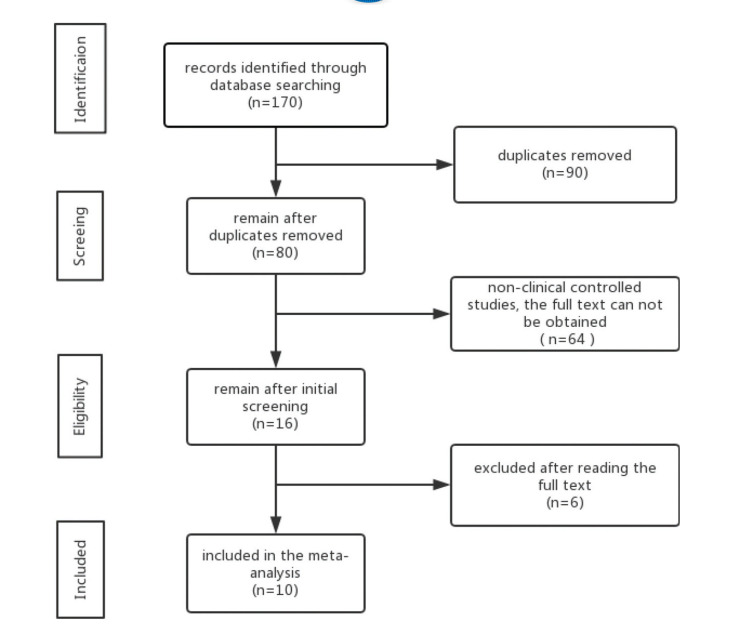
Flow chart depicting data selection and inclusion criteria used.

In total, 170 studies from various databases were initially identified, and 90 of these were then excluded as duplicates. After screening the titles and abstracts of the remaining 80 studies, 64 were excluded as they included non-clinical data, or there was no access to the full text. The full texts of the remaining studies were then screened resulting in the exclusion of an additional six studies that did not provide any data on the association between anti-*H. pylori* treatment and IBS clinical remission rates or because they were systematic reviews/meta-analyses. This left us with ten studies (6-15) that were eligible for meta-analysis, this criteria is summarized in Fig. 1. We extracted the first author's name, year of publication, IBS Rome diagnostic criteria, diagnostic criteria for *H. pylori* infection, the number of patients in the experimental and control groups, and the effective clinical remission rate for each group. These data are summarized in Table 1.

### Quality evaluation and Risk-of-bias assessments

**Figure 2:**
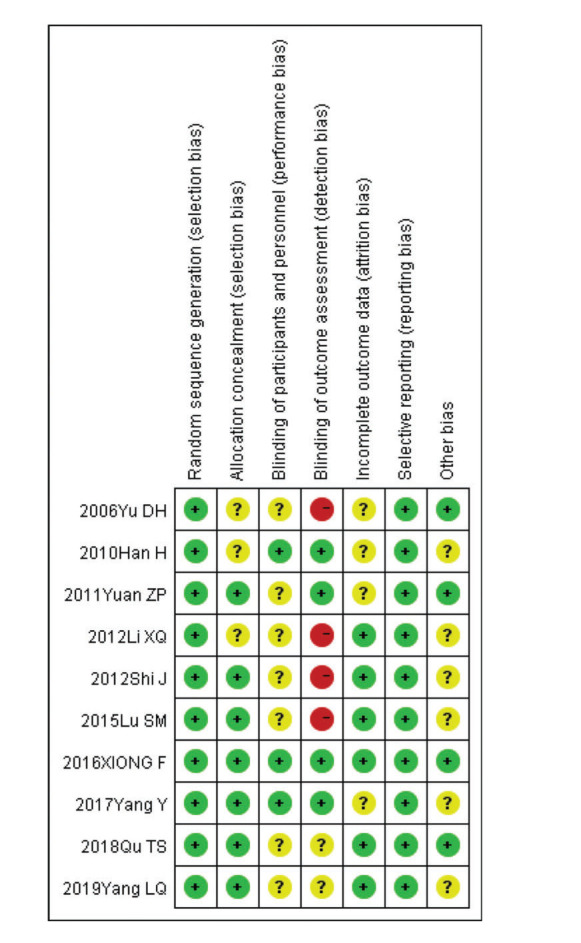
Risk-of-bias summary.

The Newcastle-Ottawa Scale (NOS) was used to evaluate the data quality in all ten of the included studies, all ten studies showed a score ≥6 points (Table 1). At the same time, two researchers (YX, LL) independently evaluated the quality of the included studies using the Cochrane risk-of-bias criteria. Every quality item was classified into unclear risk, low risk, or high risk. Any disagreements were resolved through discussion or consultation between the two independent researchers, and seven criteria were used to assess bias. Figure 2 summarizes the quality of the included literature and showed that all ten studies are relatively reliable.

### Statistical analysis

RevMan 5.3.5 and Stata 15.1 software were used to perform the meta-analysis. First, the I^2^ test was used to evaluate the heterogeneity between the studies. I^2^>50% indicated that the studies were significantly different, and I^2^<50% indicated less heterogeneity. Second, a random-effects model was used to pool the odds ratios (ORs) for each evaluation using a 95% confidence interval (CIs). The combined OR values and 95% CIs were calculated and visualized using a Forrest plot. If *p*<0.05, the difference was considered significant. Then a funnel plot was used to calculate the insecurity factor, and the publication bias was quantitatively evaluated using RevMan 5.3.5 and Stata 15.1 software. Finally, the stability of the evaluation results was evaluated using a sensitivity analysis.

## RESULTS

### Combined effect analysis

**Figure 3:**
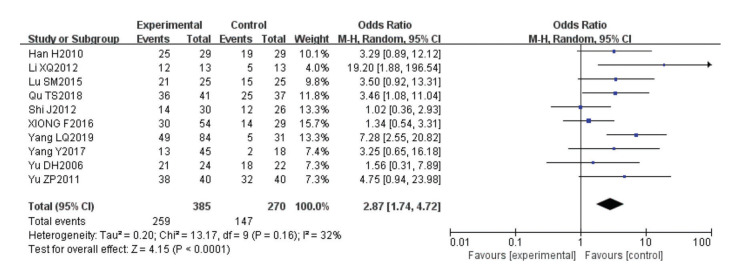
Forest plot.

A random-effects model was used to determine whether there was a significant difference in the effective remission rates for IBS patients with or without anti-*H. pylori* treatment. As shown in Figure 3, the I^2^ value was 32% suggesting a low degree of heterogeneity. The combined OR was 2.87 (95% CI: 1.74-4.72), *p*<0.0001, suggesting that anti-*H. pylori* treatment can effectively improve the clinical remission rates of IBS patients.

### Publication bias analysis

**Figure 4:**
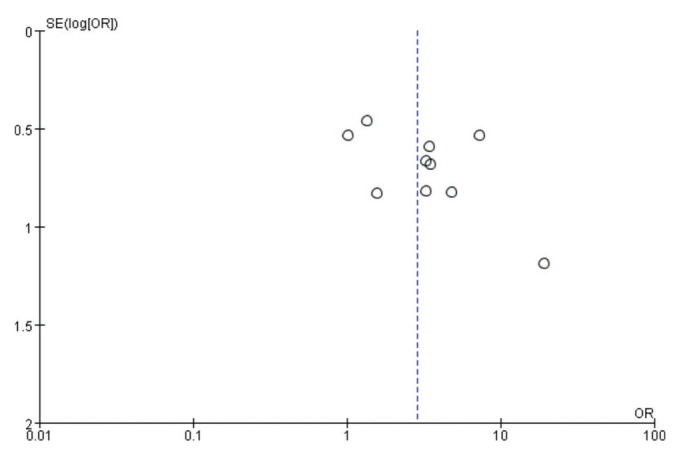
Funnel plot.

**Figure 5:**
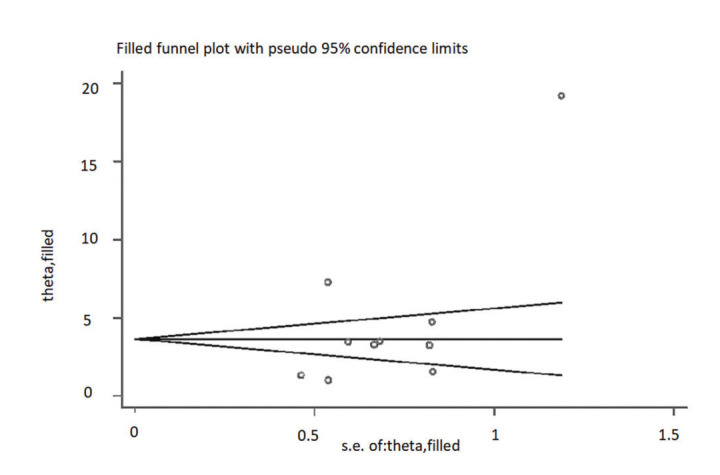
Begg’s funnel plot.

The publication bias was roughly evaluated using a funnel plot analysis. The graphs on both sides of the funnel plot were symmetrical, suggesting that publication bias had little impact on the results. To plot the scatter plot, the OR value was used as the abscissa and the SE(log [OR]) value as the ordinate (Fig. 4). The results showed that the effect value was relatively concentrated in the middle and upper parts of the graph, with a roughly symmetrical shape, indicating that publication bias was less likely to be the cause of any differences. The Begg’s method was used to quantitatively evaluate publication bias (Fig. 5), and produced a Z value of 1.25, Pr>∣Z∣=0.210>0.05, suggesting that there was no real publication bias in this study.

### Sensitivity analysis

Sensitivity analysis was performed using a fixed-effects and random-effects model, which re-estimated the results by omitting one study to determine the effect of each individual study on the overall result (Table 2). This evaluation showed that the results of this study were relatively stable.

## DISCUSSION

A total of ten studies were included in this review, and all ten evaluated the effective remission rates of the clinical symptoms (abdominal pain, diarrhea, constipation,) in IBS patients who were receiving anti-*H. pylori* treatment and those who were not. Seven studies demonstrated a positive effect and three reported a negative result. The findings of the meta-analysis showed that anti-*H. pylori* treatment could effectively improve the clinical remission rates of IBS patients.

As one of the most common pathogenic bacteria in the digestive system, *H. pylori* infections have been shown to be closely related with the development of chronic gastritis, gastric ulcers, and gastric cancer. Various antigens and toxins produced by *H. pylori* can cause cellular vacuolization, mitochondrial dysfunction, and endoplasmic reticulum-mediated stress, all of which lead to the generation of oxidative stress, which is recognized by the immune system and activates the transcriptional upregulation of pro-inflammatory cytokines and chemokines triggering a series of inflammatory responses (16,17). *H. pylori* can also activate the immune system via the brain-gut axis and via the regulation of gastrointestinal hormones, resulting in increased secretion of gastrin, cholecystokinin, and other gastrointestinal hormones, and increased activity of intestinal smooth muscle cells. This leads to a series of intestinal symptoms including diarrhea and abdominal pain (18,19). Several studies have shown that anti-*H. pylori* treatment can result in a change in intestinal flora often significantly decreasing the abundance of *Escherichia coli,* while increasing that of *Klebsiella* and other Enterobacteriaceae (20,21). But whether such changes aggravate or improve the clinical symptoms for IBS has not been evaluated.

IBS is a functional gastrointestinal disease with unclear pathogenesis. Some studies have shown that IBS patients may have low-grade inflammation and organ damage (22,23). Some animal studies have found that intestinal microecology imbalances and disordered intestinal flora can induce Kupffer cell proliferation and TNF-α and INF-γ expression, which may result in liver cell damage (24,25).

Studies have confirmed that the occurrence of IBS can be linked to increased activation of the gut-brain axis, high visceral sensitivity, intestinal flora changes, and increased immune activation. It is worth noting that intestinal flora changes may vary between regions and these changes are primarily characterized by significant decreases in *Bifidobacterium* and *Lactobacillus* in Chinese patients and a decrease in *Bifidobacteria* and an increase in *Bacteroides* in European patients (26,27). Small intestine bacterial overgrowth (SIBO) is a condition characterized by the translocation of the distal intestinal flora to the small intestine in response to a variety of factors; this results in the overgrowth of anaerobic bacteria, which is responsible for the manifestation of a series of clinical symptoms. Because of the overlap in clinical symptoms between SIBO and IBS, SIBO is currently under investigation as one of the potential pathogenic routes to IBS. In the meta-analysis, more than one-third of the IBS patients were found to be positive for SIBO; this is a significantly higher incidence than that observed in healthy individuals. Female sex, higher age, and IBS-induced diarrhea were all associated with SIBO in individuals with IBS (28). SIBO can alter the intestinal microenvironment by altering the inflammatory responses, regulating the immune system, intestinal mucosal permeability, and gas production, thereby validating the partial overlap between SIBO and IBS. Our study also demonstrated that antibiotic therapy can partially alleviate the clinical manifestations of IBS; this finding is in agreement with other literature, which suggests that antibiotic therapy can significantly improve the clinical symptoms of IBS patients with SIBO (29-31). In addition, several papers have demonstrated a link between SIBO and *H. pylori* infection. Patients with *H. pylori* are more likely to test positive for SIBO (32). Further, *H. pylori* eradication therapy can effectively relieve the clinical symptoms of patients with SIBO (33), which also suggests there is some correlation between *H. pylori* infection, and SIBO and IBS. Anti-*H. pylori* treatment may relieve the clinical symptoms of IBS by ameliorating SIBO. However, this aspect was not included in the original studies, suggesting a need for additional studies that use bacterial culture to objectively demonstrate these interactions. The intestinal mucosal immune system of IBS patients is characterized by a significant increase in the levels of lymphocytes (T cells) and mast cells, resulting in an increased release of trypsin and histamine leading to abdominal pain (34,35). Colon biopsies of post-infection IBS patients show that intestinal endocrine cells (ECs) increase more than five times in number, while lamina propria T cells (CD3, CD4 and CD8) double in number. Meanwhile, resident macrophage (CD68) populations are reduced by half and activated macrophage populations increase significantly. Activated macrophages produce many pro-inflammatory cytokines, including IL-1, which further stimulate intestinal ECs to produce excessive 5-HT, which may activate 5-HT mediated vagal afferent nuclei and increase the sensitivity of the emotional and autonomic neural networks in the brain. The vagus nerve is an important part of the microbiome-gut-brain axis that conveys the sensory and microbial information from the gut to the brain. The activation of inflammatory cells, the increase in pro-inflammatory factors, and the activation of the vagus nerve can lead to the development of diarrhea and visceral hypersensitivity (36). All these factors can affect the nature and duration of intestinal inflammation. Different individuals need different recovery times to restore their original balance, and eventually, some people will develop IBS (37,38).

Current studies suggest that IBS may be associated with *H. pylori* infection (39,40). A meta-analysis by L MR et al. (41) suggested that *H. pylori* infection rates in IBS patients were significantly higher than expected in a healthy population. However, a similar meta-analysis by Ng et al. (42) showed that there was no clear evidence to support an association between IBS and *H. pylori*; however this may be attributed to the smaller sample size in this study. Our results suggest that anti-*H. pylori* treatment could reduce IBS symptoms, and this may be mediated by the effects of anti-*H. pylori* treatments on gastrointestinal pH and flora changes, thereby promoting the recovery of the intestinal mucosal immune system and the gut-brain axis and restoring the regulation of the gastrointestinal hormone system (32). The specific mechanism underlying these effects is not yet clear, and further experiments are needed.

A key limitation of this is study the subjective outcome evaluation of the included literature, which fails to specify the detailed amelioration of clinical symptoms, such as abdominal pain, diarrhea, and constipation. Therefore, more accurate scales need to be used to effectively quantify the clinical outcomes. The development of these tools would facilitate stronger evidence-based conclusions in the future. It is necessary to point out the fact that the lack of *H. pylori* eradication in the experimental group is not the result of ineffective anti-*H. pylori* treatment but rather owing to the fact that the treatment was aimed at preventing infection instead of eradicating the bacteria. This qualification should be more apparent in clinical trials and the effect of a preventative treatment should be evaluated in additional clinical trials. In addition, because of the limited sample size, the clinical remission rate could not be compared between different subgroups of IBS, suggesting that these findings should be validated in larger populations.

## AUTHOR CONTRIBUTIONS

Xiong Y, Zhou X contributed to study design. Xiong Y contributed in literature search, analysys and draft writing. Xiong Y, Liu L, Zhou X, Wang R, Wen Y contributed in literature screening. Xion Y, Liu L, Zhou X contributed in data extraction

## Figures and Tables

**Table 1:** Basic information describing the studies included in the meta-analysis.

				IBS patients with Hp infection					
Year	Author	IBS Diagnostic criteria	Hp detection method	Number in experimental groups	Number in control group	The number of cases effectively alleviated in the experimental group	Number of cases of effective remission in the control group	Statistical significance	NOS
2016	Xiong F	Rome III	UBT+RUT	54	29	30	14	yes	8
2006	Yu DH	Rome II	UBT+RUT	24	22	21	18	no	6
2015	Lu SM	Rome III	UBT+RUT	25	25	21	15	yes	6
2018	Qu TS	Rome III	UBT+RUT	41	37	36	25	yes	7
2012	Shi J	Rome III	UBT+RUT	30	26	14	12	no	6
2012	Li XQ	Rome III	UBT+RUT	13	13	12	5	yes	6
2019	Yang LQ	Rome IV	UBT	84	31	49	5	yes	6
2017	Yang Y	Rome III	UBT+RUT	45	18	13	2	no	7
2011	Yuan ZP	Rome III	UBT	40	40	38	32	yes	6
2010	Han H	Rome III	UBT+RUT	29	29	25	19	yes	7

**Table 2:** Sensitivity analysis.

Sensitivity analysis	Number of articles	Z value	*p*-value	OR value(95%CI)
Random-effects model	10	4.15	<0.0001	2.87 (1.74,4.72)
Fixed-effects model	10	5.39	<0.00001	2.86 (1.95,4.20)
Random-effects model without the XIONG et al. study	9	4.55	<0.00001	3.29 (1.97,5.49)
Fixed-effects model without the XIONG et al. study	9	5.55	<0.00001	3.37 (2.19,5.17)
