# Erratum for: Anti-Helicobacter pylori treatment can effectively improve the clinical remission rates of irritable bowel syndrome: a controlled clinical trial meta-analysis

**DOI:** 10.6061/clinics/2021/e1857err

**Published:** 2021-01-18

CLINICS 2021;76:e1857err

Erratum for: doi: https://doi.org/10.6061/clinics/2020/e1857, published in 2020.

Replace the corresponding author: Yan Xiong

For: Xuchun Zhou*

